# Relationship between pathogenic *E.coli* O78-induced intestinal epithelial barrier damage and Zonulin expression levels in yaks

**DOI:** 10.3389/fcimb.2024.1456356

**Published:** 2024-09-23

**Authors:** Xiaoli Ren, Bin Shi, Zhenyu Chang, Jingbo Zhang, Shuo Wang, Ruidong Liu, Mudan Sang, Hailong Dong, Qingxia Wu

**Affiliations:** ^1^ Key Laboratory of Clinical Veterinary Medicine, Tibet Agriculture and Animal Husbandry University, Linzhi, China; ^2^ Institute of Animal Science, Xizang Academy of Agricultural and Animal Husbandry Sciences, Xizang Lhasa, China

**Keywords:** yak, pathogenicity *E.coli* O78, intestinal epithelial cell barrier, cell transfection, TEER, Zonulin

## Abstract

To explore whether the intestinal damage of yak colibacillosis resulted from the regulation of Zonulin expression by its pathogenic bacteria, the overexpression and interference plasmids of Zonulin were designed and cultured in Tranwell after cell transfection. Then qRT-PCR and Western blot were used to detect the results of cell transfection, 200 mL 1×105 CFU/mL *E.coli* O78 was added for 4 hours, transmembrane resistance was measured by transmembrane resistance meter, FD4 fluorescence concentration in the lower chamber was detected by enzyme labeling instrument, bacterial translocation was measured by CFU counting method, and epithelial mucin (MUC1, MUC2) and tight junction protein (FABP2, Occludin, ZO-1) were detected by qRT-PCR.

**Results:**

The Zonulin gene overexpression and knockout cell lines were successfully constructed, the TEER value of the barrier of Zonulin overexpression cell lines began to decrease at 1 h after the addition of *E.coli* O78 and reached the lowest value at 4 h, and the TEER value of Zonulin interference cell lines decreased within 1-4 h after the addition of *E.coli* O78. At 4 h, the FD4 passing capacity of Zonulin overexpression cell lines was significantly higher than that of interfering cell lines, reaching twice as much as siRNA-1. The amount of bacterial translocation in overexpressed cell lines increased rapidly within 1-4 h, and the concentration of *E.coli* in the lower chamber was significantly higher than that in the siRNA-1 group at 4 h, but there was no significant change in the siRNA-1 group in the 1-4 h. There was no significant change in the mRNA level of MUC1 in Zonulin overexpression and interference cell lines after the addition of *E.coli* O78. In the overexpression group, the mRNA levels of MUC2, Occludin, and ZO-1 were significantly decreased, and the mRNA level of FABP2 was increased considerably. These results suggest stimulate epithelial cells to secrete Zonulin protein. Many Zonulin proteins regulate the opening of tight junction structures, reduce the transmembrane resistance of the cell barrier, and improve the permeability of the cell barrier and the amount of bacterial translocation.

## 1 Introduction

Yak is an endemic breed of cattle on the Qinghai-Tibet Plateau, which occupies a dominant position in Xizang’s animal husbandry and is an important part of livestock in this area (Liu et al., 2023). Yak colibacillosis is an infectious disease caused by *E.coli* (*Escherichia Coli, E.coli*) infection, showing typical symptoms such as high fever and diarrhea. The morbidity and mortality of the disease are very high, especially affecting calves and female yaks. Diarrhea in calves and yaks infected with pathogenic *E.coli* is considered to be one of the main diseases. Diarrhea occurs in sick calves 3-5 days after birth and 3-10 days after weaning. Severe dehydration and electrolyte imbalance caused by diarrhea are one of the important causes of calf death, with a fatality rate of more than 90% (Hong, 2015; Raz, 2013; Wang et al., 2022). In recent years, Escherichosis coliform in yak has brought great challenges to the yak breeding industry. The growth retardation and death of diseased yaks are the direct causes of economic losses of herdsmen, and diarrhea yaks can also cause the spread of pathogens in the environment, making it difficult for treatment (Rehman et al., 2017). At present, the conventional method for the treatment of colibacillosis in yaks is to use antibiotics. Long-term use of antibiotics will cause problems such as drug residues and intestinal flora imbalance ( (Dong et al., 2020; Jia et al., 2018). At the same time, due to the complex physiological structure of the gastrointestinal system of ruminants, antibiotic treatment alone is not enough to solve gastrointestinal flora-related diseases. Probiotics are defined as living microbial supplements that have a beneficial effect on the host by improving the composition of intestinal microorganisms (Majidi-Mosleh et al., 2017). The most commonly used probiotics are *Lactobacillus acidophilus*, *Lactobacillus plantarum*, *Lactobacillus bulgaricus*, *Lactobacillus casei*, *Lactobacillus helveticus*, *Lactobacillus saliva*, *Bifidobacterium*, *Enterococcus faecium*, *Enterococcus faecalis*, *Streptococcus thermophilus* and so on (Hossain et al., 2012). *Lactobacillus* (*Lactobacillus, LAB*) is a Gram-positive, lactic acid-producing thick-walled bacteria, which is the most common probiotic in the intestinal tract of mammals. The efficacy of lactic acid bacteria in improving bacterial diarrhea has been widely recognized, lactic acid bacteria are members of the normal microbiota in the intestine, which work together with other microorganisms in the intestine to defend against pathogenic bacteria, improve the intestinal flora, thereby alleviating bacterial diarrhea, and also reduce the possibility of pathogen transmission due to feces during yak breeding (Sadowska et al., 2010; Vinderola et al., 2007). Studies have shown that feeding lactic acid bacteria plays a beneficial role in increasing animal meat production and reducing bovine diarrhea (Kober et al., 2022). The detection of intestinal pathogens after feeding lactic acid bacteria showed that probiotics reduced the number of pathogenic bacteria by increasing the number of beneficial bacteria in the intestinal environment and inhibiting them (Fooks and Gibson, 2002; Liu et al., 2022). It can effectively reduce intestinal pathogens and balance animal intestinal flora. It is reported that after *LAB* feeding, the content of many kinds of beneficial bacteria in the intestinal tract of calf yak was higher had higher terpenoid and polyketone metabolism, and showed an inhibitory effect on some viruses at the same time (Kobayashi et al., 2017).

The intestinal barrier is a kind of defensive structure, that separates the aseptic tissue in the body from the microflora *in vitro*, and is the first line of defense of the intestinal tract against external pathogenic bacteria, its core mechanism is the selective permeation function (Felipe-Lopez et al., 2023; Rogers et al., 2023). This selective infiltration ensures the normal digestive function of the intestine and blocks the possibility of pathogens entering the tissues of the body. Microscopic scientists in the 19th century observed and defined the paracellular space between adjacent epithelial cells. Now this intercellular structure is called the intercellular junction complex, which consists of tight junctions (TightJunctions, TJ), adhesive junctions (Adherens Junctions) and desmosomes (Desmosomes) (Cereijido et al., 2004; Farquhar and Palade, 1963). It has been found that the main function of tight junction is to maintain the surface polarity of intestinal epithelial cells and reversibly prevent the spread of macromolecules and microorganisms inside and outside the epithelial cells (Kucharzik et al., 2001; Yu and Yang, 2009). Tight junction proteins mainly include transmembrane proteins Occludin, tight junction proteins Claudin, tight junction structural proteins ZO-1, ZO-2, ZO-3, and so on. The functions of these proteins have been gradually understood. It is the binding of these protein complexes with membrane lipids that control the junction and dispersion of tight junction structures and regulate paracellular diffusion, resulting in changes in intestinal permeability (Mitic and Anderson, 1998). Blocking protein (Occludin) is the first intact membrane protein found in a tight junction. It affects the permeability of the intestinal epithelial cell barrier and regulates the entry of macromolecules (Mazzon et al., 2002; Mccarthy et al., 1996). Tight junction protein Claudin is a quadruple transmembrane protein that makes up the TJ chain, including four transmembrane domains (Transmembrane, TM), two extracellular loops containing conserved residues, and two short intracellular hydrophobic terminals (N-and C-terminals) (Tao et al., 2005) and contains 27 subtypes, and different subtypes determine the physiological characteristics of TJ. Studies have shown that the overexpression of Claudin-2 significantly increases the ionic conductivity of epithelial cells. When the expression of Claudin protein is abnormal, it usually leads to the reversible opening of tight junction structure, affecting the possibility of intestinal contents entering the intestine, and the close adhesion between cells to form permeable epithelium (Furuse et al., 2001). Subsequent studies showed that Claudin-2 formed a cation-selective paracellular pathway in addition to the tight junction permeability pathway (Amasheh et al., 2002; Colegio et al., 2002). These studies show that Claudins affect the function of TJ structure, regulate the charge of ion conductance, and form a selective intercellular pathway. The Zonula occludens (ZO) protein family, which includes ZO-1, ZO-2, and ZO-3, is a scaffold protein of TJ and belongs to the membrane-associated guanosine kinase-like protein (Maguk) family (Gonzalez-Mariscal et al., 2000). Many proteins contribute to the integrity of intestinal barriers and tight junctions. In the intestinal mucosa, the mucus layer plays an important role in stabilizing the host and microbial environment. Changes in the mucus layer or the interaction between abnormal microorganisms and the mucus layer have been proven to be the main causes of intestinal inflammation (Rokhsefat et al., 2016). The mucus layer secretes mucus hydrogel, which prevents the rapid transfer of bacteria on the surface of the intestinal barrier by adhering to the pathogen (Leoncini et al., 2024). Among them, the mucin family (Mucin, MUCs) is the main protein component of mucus on the mucous surface, which is mainly produced by goblet cells. MUC1 and MUC2 can adhere to pathogenic bacteria and protect intestinal epithelial mucosa (Cox et al., 2023; Noah et al., 2011). Changes in mucin production can weaken the intestinal mucus barrier and cause bacterial translocation and immune response, while mucosal inflammatory diseases such as ulcerative colitis are usually due to impaired expression of MUC2 protein (Boltin et al., 2013). Fatty acid binding protein (fatty acid-binding protein, FABP) is involved in the transport of intracellular fatty acids from the membrane to the membrane. After binding to FABP, fatty acids maintain their solubility and transport to the organelles (Huang et al., 2022). At present, at least 9 kinds of FABP protein family have been identified. FABP2, FABP1, and FABP9 are the three fatty acid-binding proteins specific to the small intestine (Hotamisligil and Bernlohr, 2015). Among them, FABP2 is the most abundant FABP in the small intestine, including membrane FABP2 and soluble FABP2, accounting for about 2% of intestinal epithelial cell proteins (Ockner and Manning, 1974). FABP2 is usually used as an indicator of intestinal functional integrity, and its release is affected by intestinal inflammation and intestinal microbiota (Dutta et al., 2019; Guerrant et al., 2016; Stevens et al., 2018).

Zonulin protein was originally considered a cholera vibrio toxin analog, which is related to the pathogenesis of diarrhea. In subsequent studies, Zonulin was identified as a precursor of haptoglobin 2 (Haptoglobin2, HP2). The release of Zonulin secreted by animal bodies is usually associated with diarrhea. The latest reports suggest that Zonulin is also associated with diseases such as diabetes and bipolar disorder (Sturgeon and Fasano, 2016; Wood et al., 2020), as the only known intestinal function regulator, the continuous study of its regulatory mechanism makes Zonulin protein has great potential for the treatment of diarrheal diseases. It has been reported that pathogens can increase the production of Zonulin in intestinal mucosa and combine with intestinal mucosal receptors through autocrine or paracrine, so as to improve intestinal permeability, promote intestinal inflammation, and increase the translocation ability of pathogenic bacteria (Sturgeon and Fasano, 2016).

In previous studies in our laboratory, we explored the effects of pathogenic *E.coli* on the intestinal epithelial barrier and intestinal functional proteins and proved that LAB has the ability to restore the destruction of the intestinal barrier caused by *E.coli* (Zhang et al., 2024a). Combined with their effects on the expression of Zonulin protein, we speculate that the damage of yak *E.coli* to the intestinal tract is due to the regulation of Zonulin expression by pathogenic bacteria. In this experiment, we will design Zonulin overexpression plasmid and siRNA, transfect cells, and culture TransWell to construct yak intestinal epithelial cell barrier of Zonulin overexpression and interference. After adding 200 μL 1×10^5^ CFU/mL *E.coli O78* to the treated epithelial cell barrier for 4 hours, the transmembrane resistance of the epithelial barrier was measured by transmembrane resistance meter, the bacterial translocation was measured by CFU counting method, and the FD4 fluorescence concentration in the lower chamber was detected by enzyme labeling instrument. The expression levels of epithelial mucin (MUC1, MUC2) and tight junction protein (FABP2, Occludin, ZO-1) were detected by qRT-PCR to explore whether the damage of intestinal epithelial barrier function caused by pathogens was affected by the change of Zonulin expression level.

## 2 Materials and methods

### 2.1 Preparation of pathogenic *Escherichia Coli*

Pathogenic *Escherichia coli O78* was derived from diarrheal yaks in Linzhi City, Xizang Autonomous region, and was preserved by the Clinical Key Laboratory, School of Animal Science, Xizang College of Agriculture and Animal Husbandry. The bacterial strains were resurrected, inoculated on nutrient Agar(01-023, Aobox, Beijing, China), and cultured in a constant temperature incubator(LRH-150, Shenglan, Jiangsu, China) at 37°C for 24 hours. Then a single colony was selected and put into the nutritious broth (HB0108, Hopebio, Qingdao, China) and cultured in a constant temperature shaker incubator (BSD-TF370, Boxun, Shanghai, China) at 37°C for 18-24 hours. Eosin-methylene blue Agar (HB0107, Hopebio, Qingdao, China) was used to detect bacteria, and colony forming unit (CFU) counting method was used to determine the required strain concentration.

### 2.2 Cell culture and grouping

Yak intestinal epithelial cells were isolated from 30-to 60-day-old yak embryos obtained from Nyingchi slaughterhouse. Cut the calf’s small intestine into a disposable petri dish, rinse with aseptic PBS, put the small intestine into a sterile Cillin bottle, add the appropriate amount of aseptic PBS, cut it into tissue blocks around 1 mm^3^ with aseptic scissors, and rinse with aseptic PBS. Yak intestinal epithelial cells were cultured in DMEM-F12 (Invitrogen, CA, USA) + 10% fetal bovine serum (PAN, Adenbach, Germany) in a 5% CO_2_ and 37°C incubator.

Our laboratory has used an ABC staining kit (SA1002, Boster, Wuhan, China) to detect the binding of anti-cytokeratin 18 antibody (BB12213553, BIOSS, Beijing, China), and identified the cultured cells as intestinal epithelial cells (Zhang et al., 2024b).

About 80% of the cells in the full cell bottle were digested with 0.25% trypsin (EDTA included) (25200072, Gibco, USA), and the cells were transferred to the PC membrane Transwell chamber with a pore diameter of 0.4 μm, and the number of cells was 1×10^5^/well. The culture medium was changed after 48 hours and continued to be cultured for 12 hours. The experimental groups were divided into the control group (Control group, normal cultured cells) and the model group (Model group, treated with 200 μL 1×10^5^ CFU/mL *E.coli O78* for 4 h) (Zhang et al., 2024a; Zhang et al., 2024b).

### 2.3 Construction of intestinal epithelial cell line of yak with Zonulin overexpression

#### 2.3.1 Plasmid vector construction

According to the bovine Zonulin gene sequence (NM_001040470.2) on NCBI and the restriction site on pcDNA3.1 (+) vector, primers for Zonulin were designed by Premier6.0 and synthesized by Chengdu Qingke Biological Technology Co., Ltd. The total RNA of bovine intestinal tissue was extracted and reverse transcribed into cDNA, and then PCR was performed with Zonulin gene-specific primers. The products were digested with NheI and BamHI, and the products were recovered by gel. After ligation and transformation, the clones were selected for PCR and double restriction endonuclease digestion. Sequencing comparison of positive clone liquid to Jingke Biology Co., Ltd.

#### 2.3.2 Plasmid DNA extraction

A plasmid extraction kit (D6826030000E31V014, OMEGA, USA) was used for plasmid extraction. The plasmid bacteria were resuscitated and cultured in the medium containing ampicillin antibiotics to amplify the plasmid. The bacterial solution of 50-200 mL was centrifuged at 4000 × g at room temperature for 10 min, and the bacteria were collected. The medium was abandoned and the 10 mL SolutionI/RNaseA mixture was added to the precipitation. The cells were completely re-suspended by a liquid transfer gun blowing or swirling. Add 10 mL Solution II and turn the centrifuge tube up and down 8-10 times to get a clear lysate. Add N3Buffer precooled by 5 mL and turn the centrifuge tube up and down 10 times until white flocculent precipitates are formed. The pyrolysis solution is collected by a syringe filter. ETR Solution is added to the lysate by 0.1x volume of ETR Solution, and the test tube is reversed 10 times. Then the 10 min is placed in an ice bath and 5 min is bathed in water at 42°C. At 25°C, 4000 × g centrifugation for 5 min, the Solution will form a blue layer at the bottom of the test tube. Transfer the supernatant to another new 50 mL test tube, add 0.5 times the volume of anhydrous ethanol at room temperature, gently reverse the test tube 6-7 times, and place 1-2 min at room temperature. The HiBind^®^ DNA Maxi binding column was placed in a 50 mL collection tube, 20 mL of filtrate was added to the HiBind^®^ DNA Maxi binding column, the 3 min was centrifuged by 4000xg at room temperature, and the filtrate was discarded. Re-insert the column, add 10 mL HBC Buffer, centrifuge for 3 min at room temperature 4000 × g, and discard the filtrate. The combined column was re-inserted, 10 mL DNA Wash Buffer was added, 4000 × g 3 min was centrifuged at room temperature, and the filtrate was discarded. The highest speed centrifuge dries the binding column matrix for 10 min. Place the HiBind ^®^DNA Maxi binding column on a clean 50 mL centrifuge tube, add 1-3 mL Endo-Free Elution Buffer directly to the HiBind ^®^DNA Maxi binding column matrix (the amount added depends on the expected end product concentration), and rest the 5 min at room temperature. 4000 × g centrifugal 5 min was used to elute DNA. Discard the column and store the DNA at -20°C.

#### 2.3.3 Cell transfection

Yak intestinal epithelial cells in the logarithmic growth phase were cultured in 6-well plates, and lipo3000 transfection reagents were used to transfect pcDNA3.1-NC and pcDNA3.1-Zonulin liposomes when the cells grew to 80% and 90%, with 3 repeats in each group. The transfection system was configured as shown in **Table 1**. Add 2mL of complete medium to each well. The diluted transfection reagent and diluted DNA were mixed and placed for 15 min at room temperature and then added to the corresponding well. After 48 hours, the total RNA and protein of the cells were extracted, and the expression of the recombinant plasmid was detected by qRT-PCR and WB to verify the overexpression of Zonulin.

**Table 1:** Zonulin overexpression transfection system.

	Reagent name	Gauge/hole
Dilution transfection reagent	Opti-MEM	125μL
	Lipo 3000	7.5μL
Diluted DNA	Opti-MEM	125μL
	DNA	5μg
	P3000	10μL

### 2.4 Zonulin interferes with the construction of intestinal epithelial cell line of yak

#### 2.4.1 siRNA sequence design

According to the CDS region sequence of the Zonulin gene, Zonulin small interference RNA was designed using the siRNA online design website https://rnaidesigner.invitrogen.com/rnaiexpress/, and siRNA was synthesized by Qingke Biotechnology Co., Ltd. The siRNA sequence is shown in **Table 2**.

**Table 2:** siRNA sequence.

Gene name	Gene ID	Justice chain sense (5’-3’)	Antisense chain antisense (5’-3’)
cattle-HP-1	id:280692	GGACAUCACUCCUACUUUA(dT)(dT)	UAAAGUAGGAGUGAUGUCC(dT)(dT)
cattle-HP-2	id:280692	GACAGAAGGUACCUGUCAA(dT)(dT)	UUGACAGGUACCUUCUGUC(dT)(dT)
cattle-HP-3	id:280692	GGUUCGCUAUCAGUGCAAA(dT)(dT)	UUUGCACUGAUAGCGAACC(dT)(dT)

#### 2.4.2 siRNA transfection

The dry siRNA powder was diluted to 20 μM with ddH_2_O without RNA enzyme and stored at -20 °C after sub-packaging. Lipo3000 transfection reagent was used for cell transfection without adding P3000. The transfection system is shown in **Table 3**. Complete medium 2 mL was added to each well, diluted transfection reagent and diluted siRNA were mixed and 15 min was placed at room temperature to add to the corresponding well. After 48 hours, the total RNA and protein were extracted, and the expression of Zonulin was detected by qRT-PCR and WB to verify the effect of Zonulin interference.

**Table 3:** siRNA transfection system.

	Reagent name	Gauge/hole
Dilution transfection reagent	Opti-MEM	125μL
	Lipo 3000	7.5μL
Diluted siRNA	Opti-MEM	125μL
	siRNA-1/2/3	75pmol

### 2.5 Effects of *E. coli* on Zonulin overexpression and interference with cell barrier

#### 2.5.1 Establishment of epithelial barrier *in vitro* of yaks

After overexpressing and interfering with the construction of the yak intestinal epithelial cell line, the monolayer epithelial barrier model of cultured cells in the TransWell chamber was established, and the transmembrane resistance of the upper and lower compartments was measured every 12 hours to determine the construction of cell barrier. For the establishment of Zonulin overexpression and interference monolayer cell barrier, *E.coli O78* with 1 × 10^5^ CFU was cultured for 4 h, and the grouping names were pcDNA3.1-Zonulin and siRNA-1.

#### 2.5.2 Detection of transmembrane resistance

The transmembrane resistance of the monolayer epithelial barrier was measured by a Transmembrane Resistance Meter (MERS00002, Merck, Germany). The cells successfully constructed with Zonulin overexpression and interference were inoculated into the Transwell chamber of 0.4 μm PC membrane. After 48 hours of incubation, the corresponding concentration and dose of *E.coli* O78 were added according to the experimental group in 2.2 above at 60 hours. The transmembrane resistance of intestinal epithelial cell barrier in different treatment groups was measured by a transmembrane resistance meter, and a TransWell with only culture medium was set as a blank control to measure the level of empty resistance. After the instrument was calibrated, the short electrode was inserted into the upper chamber of each group and the long electrode was inserted into the lower chamber of each group, and the readings were made on the transmembrane resistance meter. The change of transmembrane resistance during 1-4 h was recorded. The measured readings are processed and the final transmembrane resistance = (measurement group reading-blank group reading) × surface area is calculated.

#### 2.5.3 FD4 permeability detection

The permeability of the epithelial barrier was evaluated by FD4 throughput. The methods are as follows: the cells successfully constructed with Zonulin overexpression and interference were inoculated into the Transwell chamber of 0.4 μm PC membrane, the medium was changed after 48 hours of culture, and the cells were treated with *E.coli O78* at the 60th hour. After the addition of *E.coli O78*, the upper chamber culture medium of the intestinal epithelial barrier model of different treatment groups was replaced with the medium containing 20 μg/mL FITC-Dextran (4KD), and the lower chamber culture medium was replaced by aseptic PBS. After being cultured for 4 hours in the constant temperature incubator of 37°C and 5% CO_2_, the lower chamber PBS was collected and the relative fluorescence intensity across the cell barrier was measured under 485 nm excitation wavelength and 538 nm emission wavelength by fluorescence enzyme labeling instrument. The concentration of FD4 was calculated according to the standard curve.

#### 2.5.4 Detection of bacterial translocation

The number of *E.coli O78* crossing the cell barrier into the lower chamber was calculated by the CFU counting method. The methods are as follows: the cells successfully constructed with Zonulin overexpression and interference were inoculated into the Transwell chamber of 0.4 μm PC membrane, and the yak intestinal epithelial cell barrier model was constructed and treated with *E.coli O78* in the same way. At the 4th hour after the addition of *E.coli O78*, the lower chamber culture medium of TransWell was collected for bacterial CFU count. The lower chamber culture medium diluted by aseptic PBS was used to 10^-8^, and 200 μL of bacterial PBS suspension with suitable dilution concentration (10^-5^, 10^-6^, 10^-7^) was added to the aseptic plate, and the unsolidified eosin methylene blue Agar medium at about 40°C was added to the plate, and the liquid was evenly distributed by shaking the plate. After the culture medium was solidified and incubated in a constant temperature incubator for more than 16 hours, a single colony appeared. The morphology of the colony was observed to distinguish and record the number of *E.coli O78*, *E.coli O78* concentration = colony number × dilution times × 5. And calculate the average concentration of *E.coli O78* (n= 3).

### 2.6 Effects of Zonulin overexpression and interference on the expression of barrier protein mRNA

Total RNA was extracted from the treated yak intestinal epithelial cells, and the expression levels of mucin genes (MUC1, MUC2) and tight junction-related protein genes (FABP2, Occludin, ZO-1) were detected by qRT-PCR.

### 2.7 Data processing

The statistical analysis of the data is carried out by *GraphPadPrism9.0*. Each group of experiments was repeated more than 3 times. For the data of normal distribution, the data were expressed by mean ± standard deviation. For continuous variables, the values are the median and quartile range of data with non-normal distribution. For classified variables, take the percentage value. For the comparison of the two groups, the P value is obtained by using *one-way Student t-test* test and *Mann-Whitney* nonparametric test to determine the difference between groups with normal distribution data. For multiple group comparisons, the P-value is derived by the one-way ANOVA (continuous variable) or Chi-square test (categorical variable), and then the *Bonferroni* test is used to compare the group means. For all data, *p <*0.05 was considered statistically significant.

## 3 Results

### 3.1 Overexpression of Zonulin and interference with the construction of cell line

**Figure 1:**
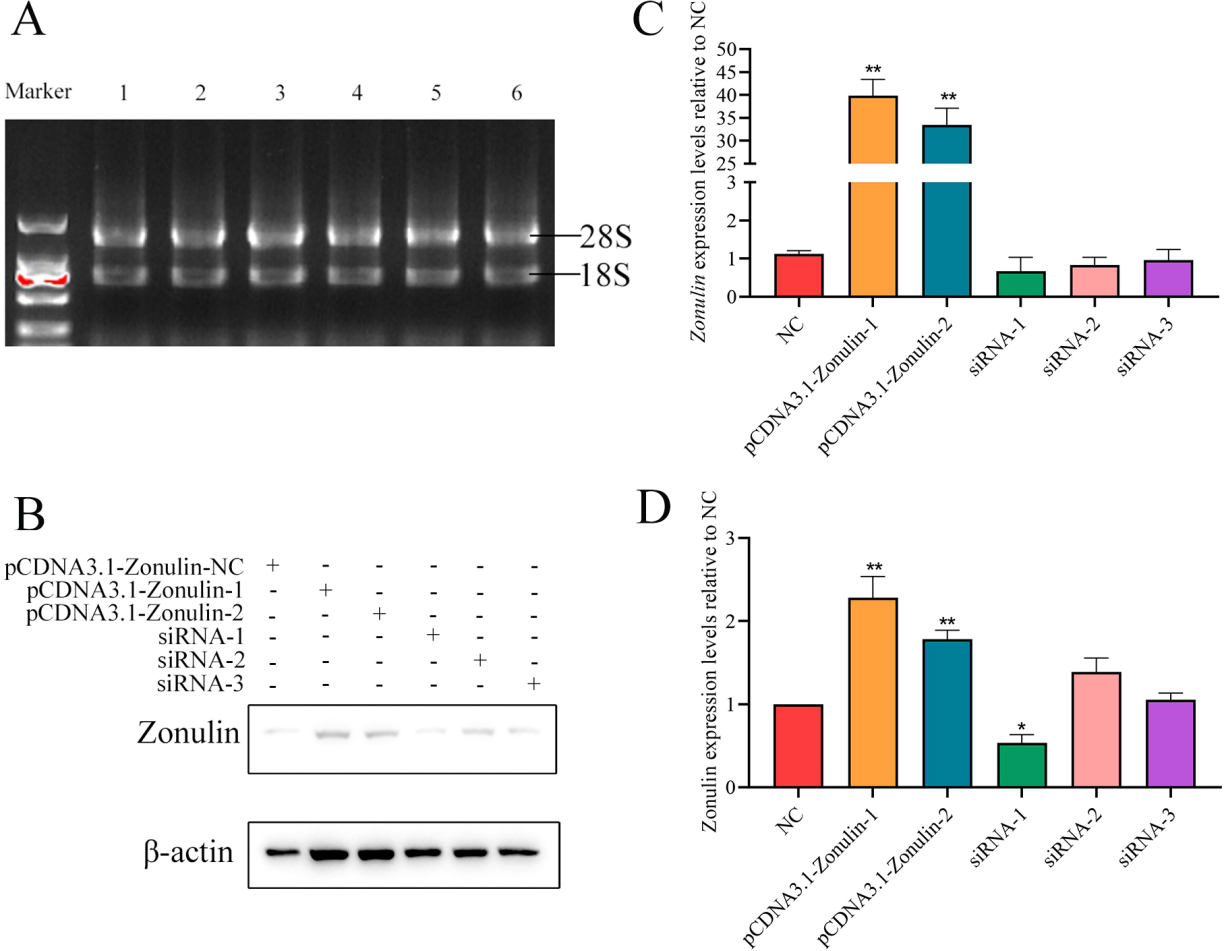
Results of Zonulin overexpression and siRNA interference efficiency detection. **(A)** RNA integrity verification. **(B)** results of WB gray value of Zonulin protein. **(C)** overexpression and interference of Zonulin mRNA relative NC expression. **(D)** relative NC expression of overexpression and interference of Zonulin protein. *p<0.05, **p<0.01.

The results of overexpression of Zonulin and interference with cell line construction are shown in **Figure 1**. After qRT-PCR detection, the expression level of Zonulin mRNA in overexpressed cell lines was more than 40 times that of the control group (**Figure 1B**). The results of interfering with the expression level of Zonulin mRNA in cell lines showed that siRNA-1 had the best effect on Zonulin knockdown. The effect of overexpression and knockdown verified by Western-blot is shown in **Figures 1C, D**. The overexpression and knockout cell lines of the Zonulin gene were successfully constructed, which were consistent with the expression level of mRNA, and the effect of siRNA-1 knockdown was the best.

### 3.2 Detection of barrier function of epithelial cells in yak

#### 3.2.1 Detection results of transmembrane resistance

**Figure 2:**
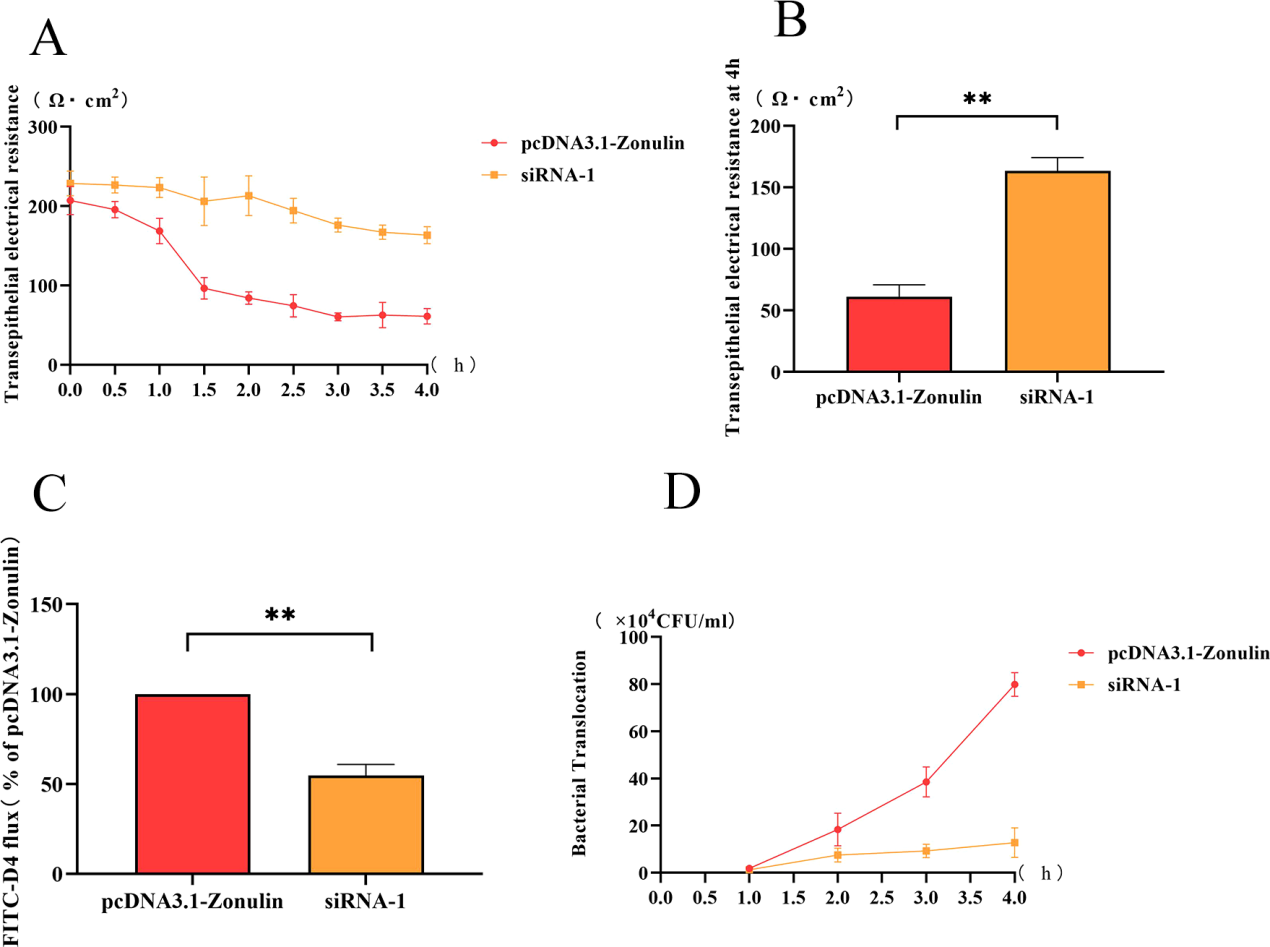
The changes of intestinal barrier function after Zonulin overexpression and interference. **(A)** *E.coli O78* affected the change of transmembrane resistance of intestinal epithelial barrier during 1-4 h. **(B)** the transmembrane resistance of intestinal epithelial barrier at 4 h. **(C)** the content of FD4 in the lower chamber at 4 h. **(D)** the amount of *E.coli* translocation changed during 1-4 h. **p<0.01.

The change of transmembrane resistance of the Zonulin overexpression cell line with time was shown in **Figure 2A**. The transmembrane resistance of the cell barrier began to decrease at 1 h, decreased to less than 100 Ω cm^2^ at 2 h, and reached the lowest value at 4 h (**Figure 2B**). The change of transmembrane resistance of the Zonulin interference cell line is shown in **Figure 2A**. The transmembrane resistance showed a downward trend in 1-4 h and decreased to the lowest value of 160 Ω cm^2^ at 4 h. At 4 h, the transmembrane resistance of Zonulin overexpression was significantly higher than that of interfering cell lines (*p* < 0.01) (**Figure 2B**).

#### 3.2.2 FD4 permeability test results

The amount of FD4 passed by the Zonulin overexpressing cell line was significantly higher than that of the interfering cell line (*p* < 0. 01), which was twice as much as that of siRNA-1 at 4 h.

#### 3.2.3 Detection results of bacterial translocation

The change of the number of *E.coli O78* in the lower chamber of TransWell at 1-4 h was shown in **Figure 2**. The bacterial translocation of overexpressed cell lines increased rapidly within 1-4 h, and the concentration of *E.coli O78* in the lower chamber reached about 8 × 10^5^ CFU/mL at 4 h, which was higher than that in the siRNA-1 group. In siRNA-1 group, the amount of bacterial translocation was less than 2 × 10^5^ CFU/mL in 1-4 h, and there was little change in the whole.

### 3.3 qRT-PCR test results

**Figure 3:**
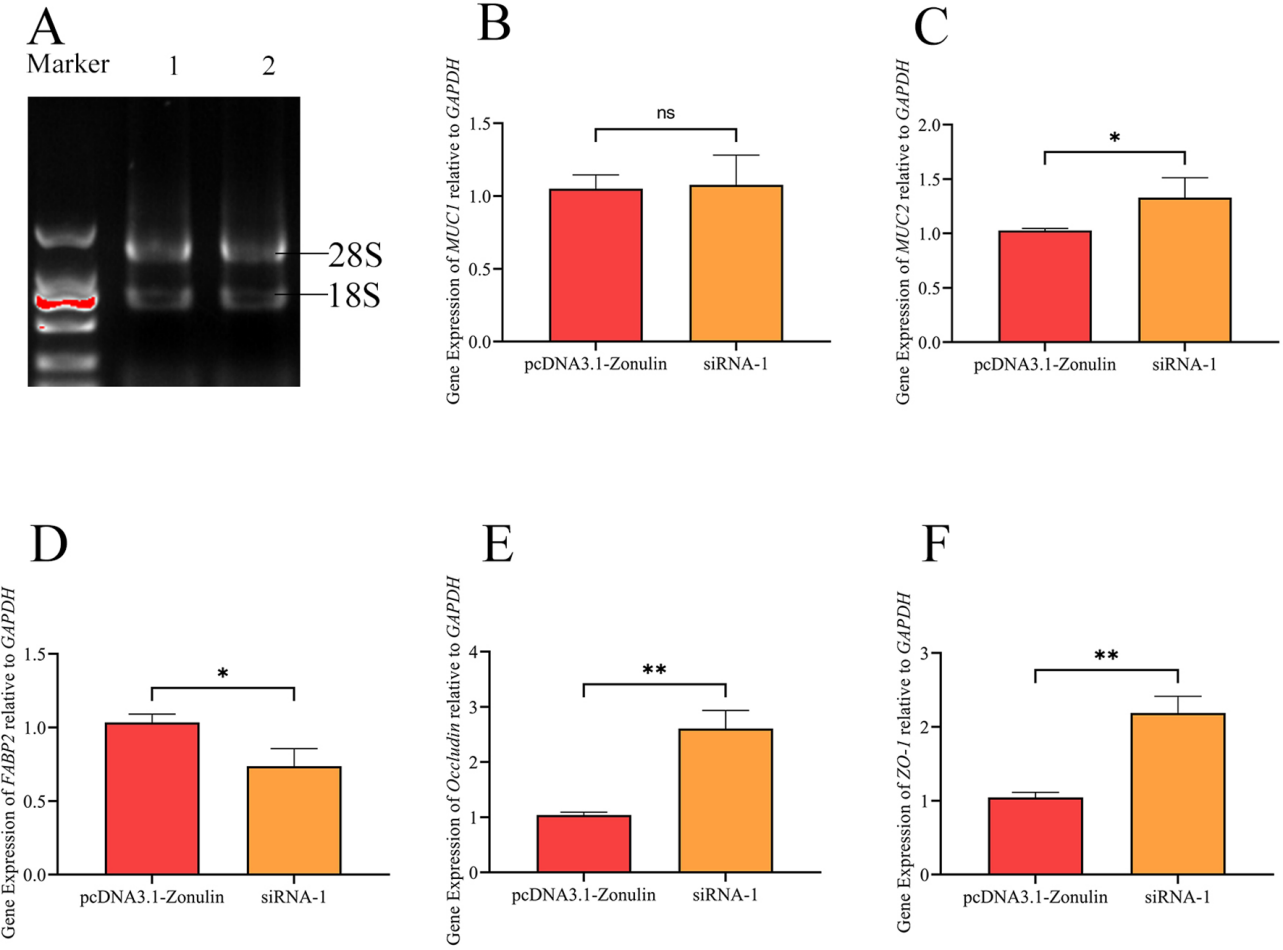
The effect of *E.coli O78* on the expression of mucin and tight junction associated protein mRNA in intestinal epithelial barrier after Zonulin overexpression and interference. **(A)** RNA integrity verification. **(B)** the relative expression of MUC1 was compared. **(C)** the relative expression of MUC2 was compared. **(D)** the relative expression of FABP2 was compared. **(E)** the relative expression of Occludin was compared. **(F)** the relative expression of ZO-1 was compared. *p<0.05, **p>0.01, ^ns^ *p* < 0.05.

The results of the detection of the Zonulin overexpression and mRNA expression levels of mucin and tight junction-associated proteins in cell lines are shown in **Figure 3**. There was no significant change in the expression level of MUC1 between the two groups, and there was no significant difference in the expression level of mRNA between the two treatment groups (*p* > 0.05), which was consistent with our previous results (**Figure 3B**). The mRNA levels of MUC2, Occludin, and ZO-1 decreased significantly (*p* < 0.05) (**Figures 3C, E, F**), while the mRNA levels of FABP2 increased significantly (*p* < 0.05) (**Figure 3D**).

## 4 Discussion

The intestinal barrier has a complex multi-layer structure, which is the physical and functional barrier between the body and the intestinal contents. The destruction of the intestinal barrier is one of the main causes of inflammatory bowel disease and diarrhea, as well as the disorder of intestinal microbiota and the translocation of harmful molecules (Camara-Lemarroy et al., 2018; Martini et al., 2017; Schumann et al., 2017). A tight junction structure is the main structure for the intestinal tract to maintain selective permeability and cellular barrier. Zonulin is a tight junction regulator, and its concentration has a great impact on intestinal permeability. Bacterial stimulation induces the release of Zonulin, which is usually accompanied by significant secretion of Zonulin, which stimulates the intestinal barrier. The induction of TJ by Zonulin may be the host’s defense mechanism. Defensive flushing is carried out through the secretion of intestinal fluid to avoid the colonization of bacteria in the small intestine (El et al., 2002). Zonulin acts through PAR2 and EGF receptors in intestinal epithelial cells, resulting in tight junction uncoupling, thereby increasing intestinal permeability (Fasano, 2011). Although diarrhea caused by the secretion of Zonulin flushes the intestinal tract to expel pathogens, the opening of TJ results in the disappearance of the selective permeability of the cell barrier, followed by the entry of bacteria into the body and the occurrence of intestinal inflammation and infection. Further stimulation of bacteria in turn continues to affect the release of Zonulin from intestinal epithelium, causing a vicious cycle of diarrhea.

In this experiment, we constructed the barrier of Zonulin inhibition and overexpression of yak intestinal epithelial cells to explore whether Zonulin gene expression affects the pathogenicity of *E.coli O78* and the regulation of tight junction-related genes. After Zonulin knock down, the effect of *E.coli O78* on intestinal barrier function was weakened. Compared with the overexpression group, FD4 permeability and bacterial translocation decreased significantly, and the transmembrane resistance of the intestinal barrier was maintained to some extent. It is suggested that in the case of overexpression of Zonulin, bacteria act on the intestinal mucous layer to destroy the intestinal barrier and stimulate epithelial cells to secrete Zonulin protein. A large amount of Zonulin protein regulates the opening of a tight junction structure, reduces the transmembrane resistance of the cell barrier, and increases the permeability of the cell barrier and the possibility of bacteria penetrating through the cell barrier. The downregulation of MUC2 represents the disappearance of the intestinal mucus layer and weakens the adhesion to bacteria. Then it brings the bacterial translocation and breaks through the barrier, which is consistent with the result indicated by the amount of bacterial translocation. We found that in the Zonulin overexpression cell line, the mRNA expression levels of tight junction proteins ZO-1 and Occludin decreased, which represented the destruction of the tight junction structure of the epithelial barrier. The down-regulation of mRNA of the constituent protein ZO-1 led to the instability of the tight junction structure, and the subsequent down-regulation of Occludin indicated the destruction of tight junction function, which was consistent with the previously reported functional study of Zonulin (Camara-Lemarroy et al., 2020). FABP2 is the most abundant fatty acid-binding protein in the small intestine (Hotamisligil and Bernlohr, 2015). Our study found that the overexpression of Zonulin relatively caused the increase of the expression of FABP2, which proved that there was a positive correlation between Zonulin and FABP2, which was also directly related to the overgrowth of bacteria in the small intestine, which was consistent with the results of previous studies (Zhang et al., 2024a). In contrast to overexpression, Zonulin knockdown maintained the mRNA expression of ZO-1 and Occludin proteins at the same time, which represented the integrity of tight junction structure and normal function of tight junction protein. It has been reported that inhibition of Zonulin can protect the tight junction of the intestinal barrier from pathogen exposure and inhibit activation of astrocytes (Zhao et al., 2023), and the use of Zonulin antagonist on respiratory epithelial cells significantly maintained the abundance and distribution of tight junction protein and the stability of transmembrane resistance (Kalsi et al., 2020). Our knockdown of Zonulin also maintains the mRNA expression of key tight junction proteins and protects the normal function of the cellular barrier.

In previous experiments in our laboratory, we explored the protective effect of *LAB* on intestinal barrier damage caused by *E.coli* O78 and obtained that LAB can regulate the expression of Zonulin, control the stability of the intestinal barrier, and protect the tight junction structure destroyed by *E.coli O78* (Zhang et al., 2024a). Similarly, the knockdown cell line of Zonulin also has the ability to resist the damage of the intestinal barrier caused by *E.coli O78*, while controlling the integrity of the intestinal tight junction structure and normal function. Combined with the results of previous experiments, we speculated that *E.coli O78* contact with the cellular barrier will cause the release of Zonulin, followed by the loss of intestinal tight junction function and the increase of cellular barrier permeability. At the same time, *E.coli O78* can destroy the mucus barrier, resulting in bacterial translocation and increased barrier permeability. *Lactobacillus Lac-2* could regulate the release of Zonulin from the intestinal epithelial barrier of yaks and reduce the stimulation of *E.coli O78* to the intestinal barrier. When the secretion of Zonulin decreased, *E.coli O78* could not cause the destruction of intestinal tight junction-related functions. At the same time, *Lactobacillus Lac-2* could compete with pathogens and had antibacterial ability, which affected the activity and translocation of *E.coli O78*, and reduced the number of *E.coli O78* translocation through the barrier. It also reduced the damage of *E.coli O78* to the mucus layer, so we observed the up-regulation of MUC2 and the decrease of bacterial translocation. From the above results, we speculate that *LAB* can restore the intestinal epithelial barrier damage caused by *E.coli O78*. On the one hand, its function is to reduce the opening of tight junction structures caused by Zonulin regulation by down-regulating the expression level of Zonulin. On the other hand, the competition and inhibition of *LAB* on *E.coli O78* growth reduced the damage of the barrier mucus layer caused by *E.coli O78*. The combined effects of the two aspects resulted in the increase in transmembrane resistance, the decrease in bacterial translocation, a decrease in barrier permeability, the increase in mucin expression level, the increase in tight junction protein expression level, the decrease in Zonulin protein expression level and the decrease in FABP2 protein expression level after *LAB*. From the results, we think that Lactobacillus Lac-2 from yak can recover the damage of the intestinal barrier caused by *E.coli O78*, and it is expected to be an effective microbial intervention therapy for E.coli disease in yaks. However, the results of our study only show that yak-derived *E.coli* O78 causes the destruction of the intestinal barrier by affecting the changes of intestinal Zonulin protein levels, and the mechanism of the intestinal barrier disruption caused by the restoration of *E.coli* O78 by yak-derived Lactobacillus Lac-2 is still unclear and needs to be further explored.

In conclusion, *E.coli* O78 from yak can cause damage to the intestinal barrier by influencing changes in the level of Zonulin protein in the yak gut, including downregulation of tight junction protein expression, disturbance of intestinal microbiota, and increased intestinal permeability. This study provides theoretical support for the mechanism of probiotics in the treatment of yak colibacillosis caused by *E.coli* O78, and has a certain effect on the improvement of yak breeding industry in Tibetan areas. Future research will explore the specific mechanism by which probiotics can achieve the effect of treating yak colibacillosis.

## 5 Conclusions

In this experiment, we constructed a yak intestinal epithelial cell line with overexpression and interference of Zonulin and investigated the effects of *E.coli O78* on intestinal barrier transmembrane resistance, FD4 permeability, and bacterial translocation, as well as the mRNA expression levels of mucin and tight junction related genes MUC1, MUC2, FABP2, ZO-1 and Occludin under the condition of Zonulin overexpression and interference. The results showed that Zonulin knockout could resist the destruction of the intestinal epithelial barrier caused by *E.coli O78*, significantly reduce the permeability of epithelial cells, maintain the integrity of epithelial barrier, significantly increase the mRNA expression level of mucin and tight junction protein, and maintain the integrity of intestinal mucosa and tight junction structure. It is proved that the damaging effect of yak-derived *E.coli* on the intestinal tract of yak comes from its regulation of Zonulin.

## Data Availability

The datasets presented in this study can be found in online repositories. The names of the repository/repositories and accession number(s) can be found in the article/supplementary material.
